# Genome-Wide Haplotype Changes Produced by Artificial Selection during Modern Rice Breeding in Japan

**DOI:** 10.1371/journal.pone.0032982

**Published:** 2012-03-13

**Authors:** Jun-ichi Yonemaru, Toshio Yamamoto, Kaworu Ebana, Eiji Yamamoto, Hideki Nagasaki, Taeko Shibaya, Masahiro Yano

**Affiliations:** 1 Agrogenomics Research Center, National Institute of Agrobiological Sciences, Kannondai,Tsukuba, Ibaraki, Japan; 2 Genetic Resources Center, National Institute of Agrobiological Sciences, Kannondai, Tsukuba, Ibaraki, Japan; 3 Genome Informatics Laboratory; Center for Information Biology and DDBJ, National Institute of Genetics, Research Organization of Information and Systems, Yata, Mishima, Shizuoka, Japan; University of Lausanne, Switzerland

## Abstract

During the last 90 years, the breeding of rice has delivered cultivars with improved agronomic and economic characteristics. Crossing of different lines and successive artificial selection of progeny based on their phenotypes have changed the chromosomal constitution of the ancestors of modern rice; however, the nature of these changes is unclear. The recent accumulation of data for genome-wide single-nucleotide polymorphisms (SNPs) in rice has allowed us to investigate the change in haplotype structure and composition. To assess the impact of these changes during modern breeding, we studied 177 Japanese rice accessions, which were categorized into three groups: landraces, improved cultivars developed from 1931 to 1974 (the early breeding phase), and improved cultivars developed from 1975 to 2005 (the late breeding phase). Phylogenetic tree and structure analysis indicated genetic differentiation between non-irrigated (upland) and irrigated (lowland) rice groups as well as genetic structuring within the irrigated rice group that corresponded to the existence of three subgroups. Pedigree analysis revealed that a limited number of landraces and cultivars was used for breeding at the beginning of the period of systematic breeding and that 11 landraces accounted for 70% of the ancestors of the modern improved cultivars. The values for linkage disequilibrium estimated from SNP alleles and the haplotype diversity determined from consecutive alleles in five-SNP windows indicated that haplotype blocks became less diverse over time as a result of the breeding process. A decrease in haplotype diversity, caused by a reduced number of polymorphisms in the haplotype blocks, was observed in several chromosomal regions. However, our results also indicate that new haplotype polymorphisms have been generated across the genome during the breeding process. These findings will facilitate our understanding of the association between particular haplotypes and desirable phenotypes in modern Japanese rice cultivars.

## Introduction

The breeding of rice (*Oryza sativa* L.) has produced new cultivars with favorable agronomic and economic characteristics such as biotic and abiotic stress resistance, high yield, and good eating quality. The semi-dwarf cultivars, such as IR8, contributed to dramatic increases in rice production from the 1960s to the 1980s, a period referred to as the Green Revolution [1]. Identification of the genes involved in disease and insect resistance has enabled the incorporation of biotic stress resistance into elite cultivars, and the grain yield of modern cultivars improved at a rate of about 1% per year from 1966 to 1995 [2], although such high gains in yield have not been achieved more recently.

Japan has a long history of breeding of *temperate japonica* rice for growth during the summer monsoon season at higher latitudes. Initially, improved cultivars were bred from landraces. Then, based on the breeding objectives of the time, consecutive efforts were made to develop new cultivars. A rapid increase in yield from the 1950s to the 1970s in Japan was achieved by adopting modern high-yielding cultivars together with intensive culture [3]. After rice production for food was improved and Japan's rice self-sufficiency approached 100%, the main breeding objective was changed from high yield to good eating quality [3]. In particular, the copious use of Koshihikari and related cultivars with good eating quality is evident in the pedigree of modern Japanese rice cultivars [4].

By crossing different lines and performing successive selection, breeders have enhanced the phenotypic performance of improved cultivars in several crop species. This process has dynamically changed the chromosomal constitution of each species by selecting and compiling a range of favorable alleles. For example, in maize, 2 to 4% of all genes have been estimated to provide evidence of artificial selection based on DNA analysis [5]. The combination of favorable alleles of these genes throughout the genome has provided a necessary variation that can be exploited to breed new cultivars.

The pedigree information for the improved cultivars has been accurately recorded, but there is little information about the chromosomal constitution of the original landraces and the improved cultivars. Insufficient knowledge of the alterations in chromosomal constitution that have occurred during the breeding process could cause problems in rice breeding, such as increased genetic vulnerability, and could prevent breeders from finding genetic solutions to problems that endanger the rice crop and to new agricultural needs.

Clarification of the chromosomal constitution of closely related populations requires the use of genome-wide markers to distinguish among the alleles revealed by these markers and to define an accession's haplotype, which consists of combinations of adjacent allelic markers. Single-nucleotide polymorphisms (SNPs) are superior to other types of markers in terms of their large number in the rice genome. Therefore, SNP markers have been widely used for studies in rice, including analyses of genetic diversity [4], [6]–[8], QTL studies [9], and marker–trait associations [10]. The use of next-generation sequencing technology has facilitated large-scale sequencing of multiple genomes, eventually leading to genome-wide discovery of SNPs [11].

The genotypes revealed by the genome-wide SNPs have revealed a deep genetic divergence between the two main varietal groups of rice (*indica* and *japonica*) [7]. Studies using four main types of DNA markers (restriction fragment length polymorphisms, random amplified polymorphic DNA, amplified fragment length polymorphisms, and simple sequence repeats) have shown that Japanese cultivars classified as *temperate japonica* had much smaller genetic diversity than those classified as *indica* cultivars [12], [13]. Based on this finding and other evidence, the Japanese rice population is believed to be genetically closed (i.e., there has been little use of foreign accessions in Japanese breeding programs), but more than thousand SNPs have nonetheless been detected and validated by means of SNP genotyping technology [4], [14], [15].

Previously, we detected and defined haplotype blocks consisting of allelic combinations of adjacent SNPs by comparing the genome sequences of two *temperate japonica* cultivars, Koshihikari and Nipponbare, and used the dynamics of these blocks to assess the change in chromosomal constitution during the modern breeding process [4]. However, the limited number of SNPs obtained from the two cultivars could not fully reveal the chromosomal constitution of other rice cultivars in the *temperate japonica* group. In the present study, we developed new SNPs and used them to analyze changes in allele frequency and haplotype diversity within a population composed of landraces and improved Japanese cultivars. The results reveal how phenotypic selection in the landraces and other breeding materials early in the breeding process has had an impact on the chromosomal constitution of modern elite rice cultivars.

## Results

### Structural changes of Japanese rice accessions following artificial selection

**Figure 1:**
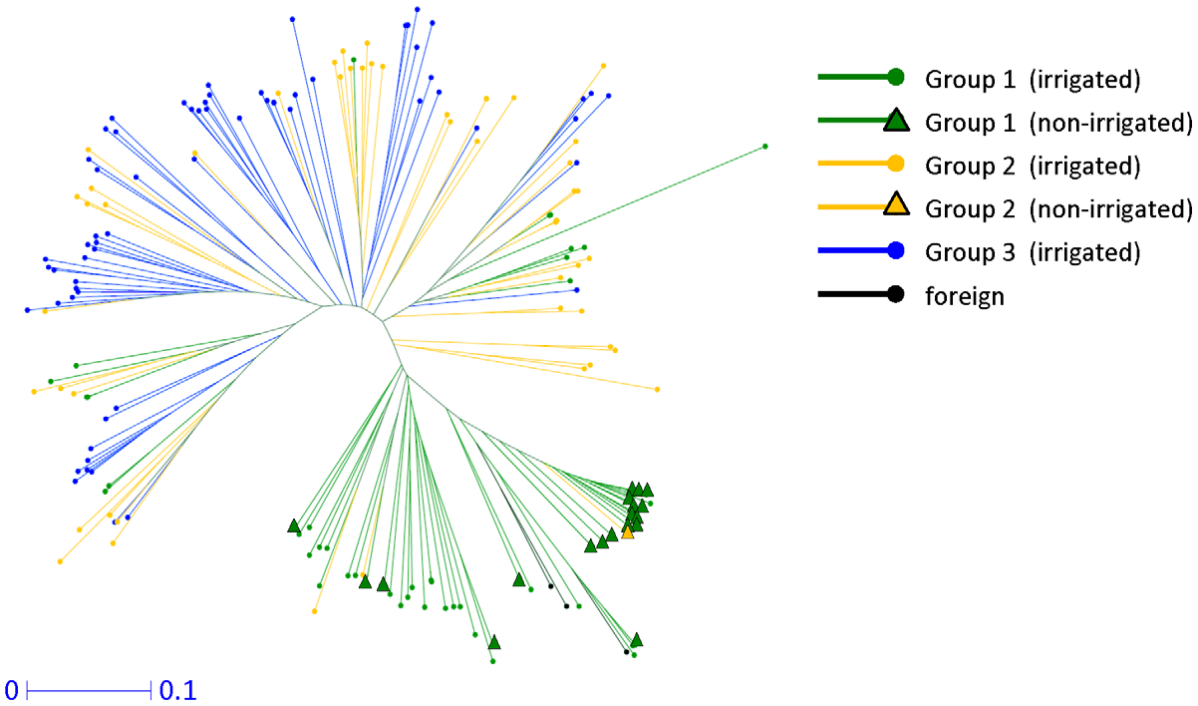
Phylogenetic relationships among the 180 rice accessions, estimated using weighted neighbor-joining analysis of 3259 SNP alleles. The horizontal bar indicates distance based on the simple matching coefficient.

**Figure 2:**
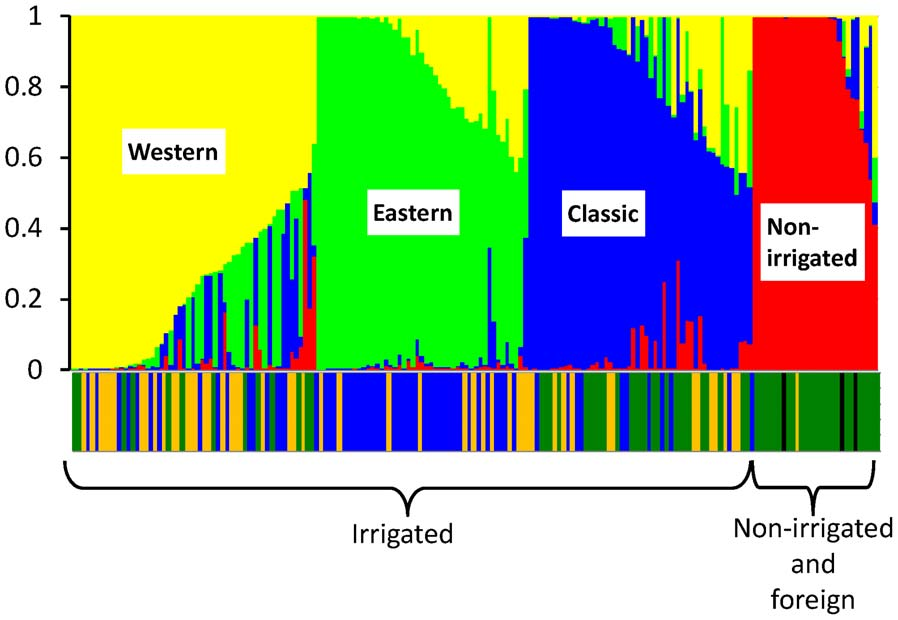
Structure analysis of the Japanese rice population using a model with four ancestral groups. In the upper part of the graph, each vertical bar represents a single accession; values displayed are the estimated membership fractions in four clusters denoted by the colors yellow, green, blue, and red. Three irrigated rice subgroups, denoted as “Western”, “Eastern”, and “Classic”, appear to be admixtures of each of the ancestral groups. The lower part of the graph indicates the breeding phase of the accessions: green, Group 1; yellow, Group 2; blue, Group 3; black, accessions categorized as “foreign” (Khau Mac Cho, Khao Nam Jen, and IR8).

To reveal the genetic relationships among the original landraces and the improved cultivars, we first classified the accessions into three groups based on the breeding phase: Group 1 (63 accessions) included landraces and cultivars bred before 1922; Group 2 (51 accessions) included cultivars bred from 1931 to 1974; and Group 3 (63 accessions) included cultivars bred from 1975 to 2005. Groups 1 and 2 contained both irrigated (lowland) and non-irrigated (upland) accessions. We then conducted a phylogenetic analysis of these accessions based on 3259 genome-wide SNPs (Figures 1, S1). Non-irrigated and irrigated rice accessions were predominantly classified into separate clusters. The genetic structure of the Japanese irrigated rice population was found to be an admixture based on the results from the InStruct software [16]. The deviance information criterion (DIC) value decreased continuously as *K* increased from 2 to 10 and an optimal *K* value therefore could not be estimated from the DIC values alone (Figure S2). Instead, we identified the most probable model as *K* = 4 because the non-irrigated and irrigated subgroups of the rice population were distinguished using *K* = 4 as the simplest cluster model (Figure 2).

We divided the Japanese rice accessions into four subgroups: Western, Eastern, Classic, and Non-irrigated. The Western and Eastern subgroups were given these names because almost all cultivars in these subgroups were bred in the western (or southern) and eastern (or northern) regions of Japan, respectively (Figure 2). The Classic and Non-irrigated subgroups were given these names because the oldest accessions (Group 1) and the non-irrigated rice cultivars, respectively, were the dominant accessions in these subgroups. The Classic subgroup also includes most of the cultivars bred in the northernmost part of Japan (Hokkaido Island). More than half of the accessions belonging to the three irrigated subgroups consisted of two or more admixed ancestors (i.e., within each of these accessions, the frequency of alleles from a single ancestor was <0.8). These admixtures accounted for 53% of the Western subgroup, 43% of the Eastern subgroup, and 42% of the Classic subgroup.

We then assessed the relationship between the four ancestral subgroups and the three breeding phase groups. We calculated the mean proportions of each of the four ancestral components (Western, Eastern, Classic, and Non-irrigated) for irrigated members of each of the three breeding phase groups and for the non-irrigated accessions. The irrigated rice groups and the non-irrigated rice group had significantly different main ancestral components (Table 1). Group 1, which contains the oldest cultivars, had the highest values for the Classic and Western components and the lowest for the Eastern component (Table 1, *P*<0.05). Group 2, which contains cultivars that are more modern than those in Group 1, had a high value for the Western component, but its lowest value was for the Non-irrigated component, in marked contrast to Group 1. Group 3, which contains the most recent cultivars, had the highest value for the Eastern component. The mean proportion of the Eastern component increased during the breeding process (from Group 1 to Group 3). Conversely, the mean proportion of the Classic component gradually decreased during the breeding process. We analyzed the change in the chromosomal constitutions during the breeding process only for the irrigated rice cultivars because of the difference in population structure between the irrigated and non-irrigated cultivars.

**Table 1:** Results of the Mann–Whitney test for the mean value of the four ancestral components (Western, Eastern, Classic, and Non-irrigated) between the three irrigated rice breeding groups (1 to 3) and the non-irrigated rice group with reference to the population structure.

	Group 1				Group 2				Group 3				Non-irrigated			
	Mean	±	S.E.1)		Mean	±	S.E.		Mean	±	S.E.		Mean	±	S.E.	
Western	0.279	±	0.055	ab2)	0.489	±	0.052	a	0.280	±	0.042	ab	0.078	±	0.034	c
Eastern	0.029	±	0.010	c	0.240	±	0.044	b	0.543	±	0.049	a	0.011	±	0.006	d
Classic	0.510	±	0.062	a	0.258	±	0.048	b	0.161	±	0.041	b	0.139	±	0.052	b
Non-irrigated	0.181	±	0.049	b	0.014	±	0.003	c	0.015	±	0.006	c	0.772	±	0.074	a

1) Standard error.

2) Values in a row labeled with different letters differ significantly (*P*<0.05, Mann–Whitney test with Bonferroni's correction).

**Figure 3:**
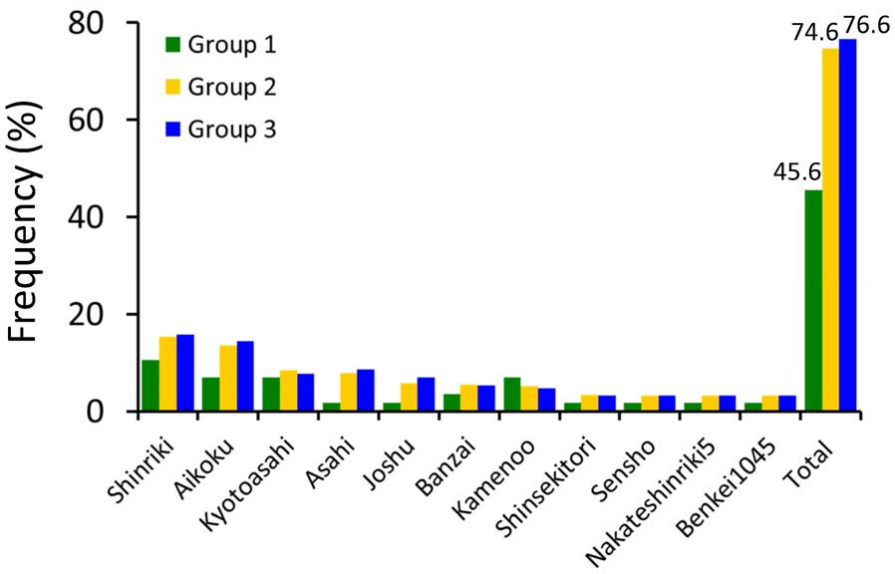
Frequencies of the 11 most common ancestral landraces in 41, 50, and 63 accessions of irrigated rice cultivars in groups 1, 2, and 3, respectively. The number of ancestral cultivars was estimated based on the pedigree data in the database of Japanese rice cultivars (http://ineweb.narcc.affrc.go.jp/). The bar “Total” indicates the total proportion for the 11 previous bars for each of the three groups and the values for the total proportion are shown above each of three bars.

The constitution of founder landraces was analyzed for 41, 50, and 63 irrigated rice cultivars in groups 1, 2, and 3, respectively (Figure 3). The frequencies of the 11 most common ancestral landraces of Japanese rice showed that these ancestries changed dramatically between groups 1 and 2 but did not change much between groups 2 and 3. In Group 1, total proportion of the 11 ancestors was less than 50% but in both Group 2 and Group 3, it accounted for 70% or more.

### Change in linkage disequilibrium and haplotype blocks

**Figure 4:**
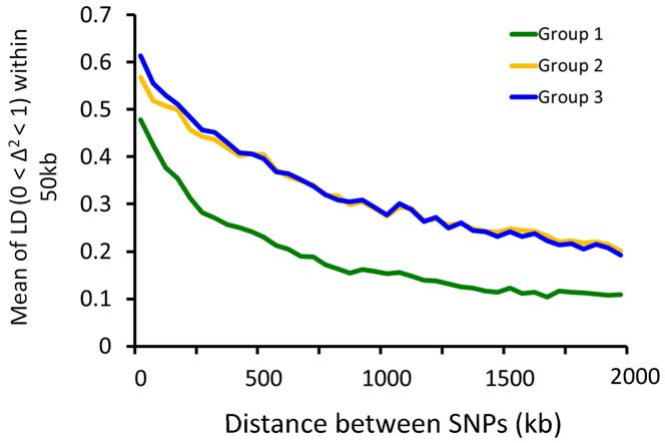
Relationships between complete linkage disequilibrium (LD, where 0<Δ^2^< 1) and the distance between adjacent SNP pairs. The mean of the adjacent SNP pairs for LD was calculated within each 50-kb interval.

We calculated the change in linkage disequilibrium (LD) by calculating the number of adjacent SNP pairs that showed complete LD (0<Δ^2^<1) in each breeding phase group, excluding the non-irrigated accessions (Figure 4). The mean LD within 50-kb intervals showed that Group 1 had a lower LD than groups 2 and 3.

**Figure 5:**
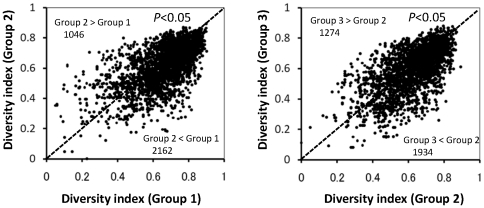
Relationships between the haplotype diversity indices for the three breeding phase groups. The diversity index for each of the 3211 haplotype blocks was calculated as [number of polymorphisms in haplotype blocks defined by a SNP allele combination in consecutive five-SNP windows (*N* = 3211)]/[number of landraces or cultivars in each group]. The total numbers of haplotype blocks that had higher diversity in one group than in the other are shown in the corners of the graphs. Three blocks showed the same values for diversity index. Significance was calculated using the Mann–Whitney test based on comparisons of diversity index values between the two groups.

We considered that the number of polymorphisms in the haplotype blocks defined by the combination of five adjacent SNP alleles might be a good representation of the genetic diversity of rice. We were able to define 3211 haplotype blocks from the 3259 SNP combinations because we found no five-SNP blocks at the end of the long arm of any of the 12 chromosomes. Figure 5 shows the comparative distributions of the diversity index in corresponding haplotype blocks (i.e., blocks at the same position) between pairs of the three breeding phase groups. Roughly two-thirds of the haplotype blocks had higher diversity indices for Group 1 than for Group 2, and the difference was significant (*P*<0.05). Thus, the diversity of haplotypes decreased between these two phases of the breeding process. Moreover, the haplotype diversity decreased significantly from Group 2 to Group 3 (*P*<0.05), although the magnitude of the decrease was lower.

### Association between the positions of QTLs or genes with phenotypic effects and the haplotype block diversity

Owing to the nature of artificial selection, individuals with specific haplotype blocks associated with favorable phenotypes might have been preferentially selected for breeding, causing a change in the frequency of the haplotype blocks in the rice population. Modern improved cultivars in Japan have been selected to have both high yield and good eating quality. Therefore, it is likely that specific haplotype blocks with extremely reduced diversity might be associated with such traits. To examine this possibility, we extracted the chromosomal positions of haplotype blocks that contained less than half the number of polymorphisms in groups 2 and 3 than in Group 1. To clarify the association between haplotype blocks and QTLs or genes, we used QTL and gene information from the Q-TARO database [17], data from our previous review [18], and data from other published studies [19]–[39].

**Figure 6:**
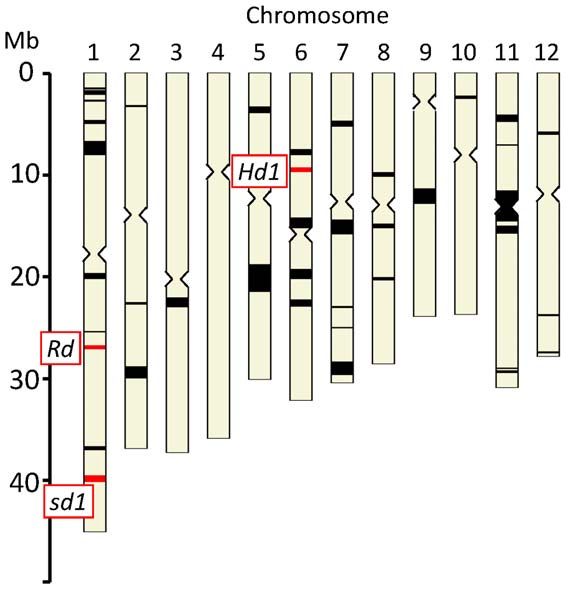
Distribution of haplotype blocks for which groups 2 and 3 had less than half the number of polymorphisms in Group 1. Non-irrigated accessions were excluded from this analysis. Red squares show haplotype blocks that were co-localized with the *Rd*, *sd1*, and *Hd1* loci. Detailed information is provided in Table S3.

Among the 3211 haplotype blocks, the diversity of 40 genomic regions corresponding to 136 haplotype blocks in groups 2 and 3 was less than half of that in Group 1 (Figure 6, Table S3). Figure 6 shows the regions in which chromosome diversity was reduced in groups 2 and 3 relative to Group 1. These 40 regions were located on all chromosomes except chromosome 4, and ranged widely in length (from 62 to 2958 kb). The largest of these 40 regions was located on chromosome 11 between positions 11.6 and 14.6 Mb (relative to Nipponbare Pseudomolecules Build 4.0 [40]), and was co-localized with the centromeric region (12.2 to 14.2 Mb) on the same chromosome. Each of the 40 regions comprised two or more haplotype blocks, with a maximum of nine. The total number of QTLs co-localized within these 40 regions was 454 when redundant information was included (Table S3). There were no obvious correlations between the number of QTLs and the length of the corresponding regions: for example, no QTL was detected near the 29-Mb position on chromosome 11, but 40 or more QTLs were observed near both the 37- and 40-Mb positions on chromosome 1.

To clarify the relationship between haplotype blocks within these regions and phenotypes, we listed rice genes that are known to affect phenotypes or functions and that were genetically proven in other studies to be co-localized with these regions. We found 20 genes [19]–[39] in these regions (Table S3), including three characterized genes that are responsible for natural variation in phenotype and other genes identified by means of transgenic or mutant analysis. The regions that were co-localized with the genes responsible for natural variation were associated with relatively large numbers of QTLs: for example, the dwarfing gene *sd1*, which was located within a 617-kb region consisting of five haplotype blocks, was co-localized with 42 QTLs, the largest number that we observed. The heading date gene *Hd1* was co-localized with 32 QTLs.

### Generation of new SNPs, and detection of new haplotype polymorphisms in modern rice cultivars

**Figure 7:**
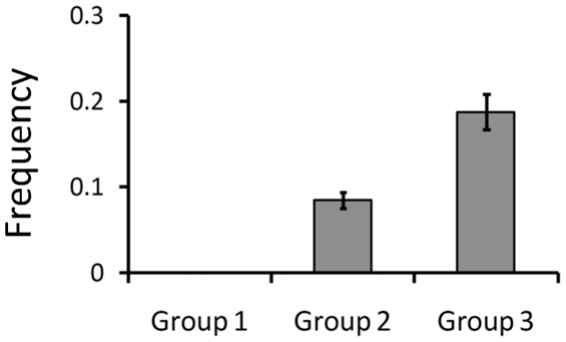
Comparison of allele frequency for the 46 new polymorphic SNPs detected in groups 2 and 3 but not in Group 1. Non-irrigated accessions were excluded from this analysis. Values are means ± standard errors. Detailed information is provided in Table S4.

**Figure 8:**
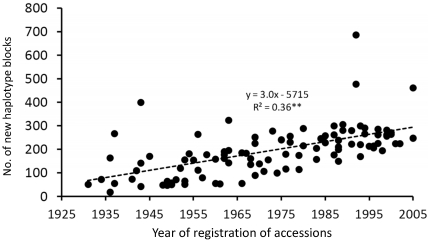
Relationship between the number of new polymorphisms in the haplotype blocks and the year of registration of the cultivars (1931 to 2005). Non-irrigated accessions were excluded from this analysis. The linear model *y* = *a*+*bx*+ε showed a highly significant regression (**; *P*<0.01).

We detected 46 new SNPs in groups 2 and 3. These SNPs were monomorphic in Group 1, but polymorphic in groups 2 and 3. The mean frequency of these SNPs in Group 2 was 0.085 (Figure 7, Table S4). In Group 3, the mean frequency of these SNPs increased to 0.189. The number of new polymorphisms within the 3211 haplotype blocks gradually increased as cultivars underwent modern breeding during the 75 years from 1931 to 2005 (Figure 8). Linear regression analysis showed a significant association between the year of registration of an accession and the number of new haplotype polymorphisms (*P*<0.01, *R* ^2^ = 0.36). The slope of the regression line indicated that an average of about three new polymorphisms had been generated each year over the 75-year period.

## Discussion

### Pedigree data and LD decay revealed a strong population structure in the Japanese rice population at the beginning of modern breeding

The discovery of a large number of genome-wide SNPs using next-generation sequencing technology has helped researchers to genetically characterize the rice genome at extremely high resolution [4], [6], [7]. We believe that the diversity of haplotypes in whole genomic regions can be represented by allelic information derived from these high-density SNPs. To define the haplotype polymorphisms with high accuracy, the length of the haplotype blocks should be smaller than the length of the LD blocks, and several measurements of LD have been reported for rice [10], [41]–[43]. The LD decay in *japonica* is generally larger than that in *indica*: >500 kb for *japonica* versus 75 to 150 kb for *indica* [41], and ∼167 kb for *japonica* vs. ∼123 kb for *indica* [10].

In this study, the LD in Group 1 at SNP intervals of 2 Mb or less was less than that in the improved cultivars in groups 2 and 3, and differed only slightly between groups 2 and 3 (Figure 4). This suggests that a strong population structure developed in the population of cultivars that have undergone modern selection breeding. In particular, the founder effect arising from the limited number of parental cultivars used during the early stages of rice breeding in Japan might have caused a loss of genetic variation.

The Japanese irrigated rice population is descended from a subset of the *temperate japonica* population of other Asian countries centuries before the start of our study period. Moreover, during the long modern history (i.e., since ca. 1910) of Japanese rice breeding, no cultivars introduced from abroad were directly used as breeding materials (Table S1). A pedigree diagram for a leading Japanese variety, Koshihikari, also showed that no foreign cultivars were directly used as founders [14]. Even when cultivars with useful properties such as blast resistance were introduced and used as donors for Japanese rice cultivars, repeated backcrosses with Japanese rice cultivars have been carried out to maintain favorable phenotypes such as eating quality. The available evidence therefore suggests that few genome segments were derived from foreign cultivars and that their influence was negligible. We believe that the low degree of genetic variation in the Japanese rice population that resulted from founder effects has not been greatly affected by introgression of genes from foreign accessions. Our pedigree analysis to compare groups 2 and 3 with Group 1 also supports our belief that most modern Japanese rice cultivars were mainly derived from crosses among a limited number of ancestral Japanese landraces (Figure 3). Moreover, the diversity of the haplotype blocks has decreased since the start of the breeding process (Figure 5).

Our results indicate that specific landraces dominated by Western genotypes, with a few Non-irrigated and Classic types, were selected for breeding of the Group 2 cultivars. Our observation of a higher frequency of haplotype blocks from the Eastern type in Group 3 than in the other groups indicates that Koshihikari and related cultivars were the main parental cultivars for this group. At present, Koshihikari is still the leading cultivar in Japan, even though it was registered about 50 years ago; in addition, its progeny—Hitomebore, Hinohikari, and Akitakomachi—are ranked 2nd, 3rd, and 4th, respectively, in the area of cultivation in Japan [4].

### Evidence of haplotype selection

Haplotype blocks with less than half the diversity in groups 2 and 3 than in Group 1 were detected within 40 chromosomal regions (Figure 6, Table S3). Reduced diversity suggests that haplotype selection has occurred in these specific regions as a result of artificial selection during rice breeding. We consider it likely that this haplotype selection was associated with the selection of particular traits of interest, such as grain yield, heading date, and eating quality.

Among the 40 regions in the present study, 3 were co-localized with “QTL cluster regions”. QTL clusters were defined as regions with 15 or more QTLs per 2-Mb interval. Such QTL clusters were found in two regions (4 to 6 Mb and 38 to 44 Mb) on chromosome 1, in one region (0 to 2 Mb) on chromosome 3, in one region (2 to 8 Mb) on chromosome 6, in one region (32 to 34 Mb) on chromosome 4, and in one region (20 to 22 Mb) on chromosome 9 in our previous study [17]. Three of these six QTL clusters were associated with regions of diminished diversity, suggesting that phenotypic selection could affect the diversity of specific haplotype blocks.

Twenty characterized genes were co-localized in the haplotype blocks that showed less diversity as a result of breeding selection (Table S3); however, only three of these are known to be responsible for natural variation. The *Rd* gene, located at position 27.0 Mb on chromosome 1, is involved in proanthocyanidin synthesis [21]. The complementary effect of *Rd* on the *Rc* gene, which encodes a basic helix-loop-helix protein, has also been demonstrated, and a homozygote with two dominant alleles for *Rd* and *Rc* has a red pericarp. In contrast, the pericarps of the *rdRc*, *Rdrc*, and *rdrc* genotypes are brown, white, and white, respectively. Group 1 includes some red rice cultivars, but groups 2 and 3 do not. Therefore, in the case of the *Rd* region, it is possible that population stratification rather than a genetic bottleneck caused by breeding selection has affected the level of diversity in the haplotype blocks. This indicates that population stratification is responsible for the decreased diversity in the *Rd* region. That is, selection for white rice may have resulted in reduced diversity in this region in groups 2 and 3.

The diversity of haplotype blocks that were co-localized with the *sd1* [32] locus also decreased during the breeding process. The mutation encoded by the *sd1* gene induces the shortening of plant height (i.e., semi-dwarf phenotype) by depressing gibberellin biosynthesis [32]. Other studies have suggested that the *sd1* allele associated with a weak dwarf phenotype has been selected and used in *japonica* cultivars, whereas the allele associated with a strong dwarf phenotype has been used in *indica* cultivars [44], [45]. A recent study supports this proposition [46]: it showed that the allele for shorter culm length was selected and used by ancient Japanese farmers and that more selection for this allele would therefore not be expected. Other QTLs or genes tightly linked with the *sd1* locus in the same haplotype blocks might also have been selected, and the number of polymorphisms within them might have decreased. For example, *OsPdk1* [27] and other unidentified genes in this region associated with a phenotype and specific allele might have been selected.

Co-localization between a low-haplotype-diversity region and a particular gene responsible for natural variation was also found in the case of the *Hd1* gene [37] at position 9.3 Mb on chromosome 6. *Hd1* plays a major role in photo-sensitivity and has multiple functional and non-functional alleles [47]; it interacts with other QTLs and genes (e.g., *Hd2*, *Hd3a*, *Hd5*, and *Hd6*) to control heading date [48]–[51]. Recently, DNA sequence information relating to natural variation of heading date has been clarified in both genic [52] and adjacent [53] regions of *Hd1*, but there is insufficient information about the relationships between variation of alleles and gene function except in the case of small deletions of genic regions. The reduction of haplotype diversity near *Hd1* is likely to be caused by phenotypic selection in the direction of early or late heading, depending on the location where the breeding took place.

Many regions with low haplotype diversity were not associated with QTLs or genes. This finding may be explained by the existence of undiscovered genes affecting important traits in Japanese cultivars. Alternatively, haplotype frequency might have been accidentally skewed for unknown reasons. For example, the genetic bottleneck associated with the small number of ancestors used to breed modern cultivars might have led to the presence of random regions with very low diversity due to a strong genetic drift effect. We detected a decrease of haplotype diversity from Group 2 to Group 3, but the genomic positions encompassing the genes related to eating quality and other traits could not be determined (data not shown). The decrease of haplotype diversity may have resulted simply from repeatedly using specific cultivars such as Koshihikari and its relatives. This hypothesis is supported by the higher Eastern component in Group 3 (Figure 2) than in Group 2 and by the pedigrees of modern rice cultivars [4].

Further analysis will be required to determine whether there is an association between a reduction of haplotype diversity in specific regions and the selection of a particular target trait during the breeding process.

### New polymorphisms observed in haplotype blocks

In general, artificial selection results in new combinations of traits of interest. Therefore, we speculated that new polymorphisms of haplotype blocks must have been generated by the modern breeding process. Although our results indicate that the diversity of haplotypes decreased gradually during the modern breeding period (Figure 5), new polymorphisms have been generated during the modern breeding process (Figure 8). Considering that it takes about 10 years to develop and release a new cultivar, and given our estimation of 3 new polymorphisms per year (Figure 8), we estimate that about 30 new polymorphisms were generated per cultivar. The generation of new polymorphisms can be attributed to three processes: the appearance of new SNPs, the recombination of existing haplotype blocks, and a combination of these two processes. The frequency of the 46 new SNPs that were present in groups 2 or 3 but not Group 1 increased from Group 2 to Group 3 (Figure 7). It is possible that the haplotypes containing these SNPs will be useful for breeding. It would be interesting to perform detailed analysis of the association between such chromosomal regions and traits of interest. Performing haplotype definition of current and old cultivars that are potential parental lines for breeding will contribute to the design of parental combinations and enable selection for superior combinations of haplotype blocks.

## Materials and Methods

### Plant Materials

We used a collection of 177 accessions, including landraces and improved cultivars, plus 3 foreign cultivars (IR8, Khao Mac Kho, and Khau Nam Jen) in this study (Table S1). A total of 149 landraces and improved cultivars were selected from our previous study [4]. We included an additional 28 accessions comprising non-irrigated (upland) and irrigated (lowland) landraces that had been analyzed previously in a study of Japanese rice diversity [14]. To assess the change in genomic constitution within and between the various breeding phases in Japanese history, we classified the 177 accessions into three groups: Group 1, landraces and cultivars developed before 1922 (63 accessions); Group 2, cultivars developed from 1931 to 1974 (51 accessions); and Group 3, cultivars developed from 1975 to 2005 (63 accessions). This classification was based on the categories used in a previous study [54]. Non-irrigated rice accessions accounted for 22 of the 63 accessions in Group 1 and 1 of the 51 accessions in Group 2. Group 3 included no non-irrigated accessions.

### Selection of SNPs for genotyping

We allocated 2688 genome-wide SNPs derived from a comparison between Koshihikari and Nipponbare [4] to three 768-plex sets and one 384-plex SNP set for analysis using the GoldenGate BeadArray technology platform (Illumina Inc., San Diego, CA, USA). In addition, we selected two 768-plex SNPs sets derived from a comparison between the reference cultivar Nipponbare and Rikuu132 or Eiko, respectively, to cover chromosomal regions in which few SNPs have been detected between Koshihikari and Nipponbare [14].

The total set of 4224 SNPs was verified as true SNPs by genotyping of the 180 accessions. For the SNP verification, we obtained DNA extracts from a piece of leaf blade of one individual of each accession, and used 5-µL aliquots of 50 ng/µL total DNA for analysis using the Bead Station 500G system (Illumina). All experimental procedures for the SNP typing followed the manufacturer's instructions.

For each accession, heterozygous alleles were treated as giving no signal. We adopted the following criteria for the classification of a SNP as non-informative: (1) no information on its genome position (International Rice Genome Sequencing Project Pseudomolecules Build 4; [40]), (2) heterozygous alleles or no signals detected in more than 50% of the accessions, and (3) an allele frequency of ≤2% (to minimize the risk of genotyping error). Based on these criteria, we selected a total of 3259 informative SNPs derived from Koshihikari (2093 SNPs), Eiko (493 SNPs), and Rikuu132 (673 SNPs) and used them for analysis of the genomic constitution of the Japanese rice population (Table S2, Figure S1).

### Phylogenetic and structural analysis

A phylogenetic tree for the 180 rice accessions was drawn based on the genotypes defined using the 3259 SNPs using the weighted neighbor-joining method with simple matching coefficients implemented in the DARwin software ([55]; http://darwin.cirad.fr/darwin). The population structure of the Japanese rice population was analyzed using the InStruct software ([16]; http://cbsuapps.tc.cornell.edu/InStruct.aspx) with the admixture model; InStruct offers the advantage of not requiring Hardy-Weinberg equilibrium in the population being studied. The run-length parameters used in InStruct were 100 000 burn-in iterations, and 200 000 replications per chain after the burn-in period using the Markov-chain Monte Carlo method. We used simulations with *K* values ranging from 2 to 10, with five replications, to calculate the DIC value. The optimal *K* value can be chosen based on the minimum DIC that provides a distinct population structure. On this basis, we chose *K* = 4 (four ancestral groups) for the Japanese rice population. To assess the relationship between these four ancestral subgroups and the four cultivar groups (the three irrigated rice accession groups in groups 1, 2, and 3 and the non-irrigated rice group in groups 1 and 2), we calculated the mean proportions of each of the four ancestral components and statistically analyzed the results using the Mann–Whitney test with Bonferroni's correction for each of the four cultivar groups.

### Pedigree analysis

To demonstrate the number of ancestors used in modern Japanese rice cultivars, we analyzed the pedigrees of 41, 50, and 63 cultivars in Group 1, Group 2, and Group 3, respectively, based on the pedigree data in the database of Japanese rice cultivars (http://ineweb.narcc.affrc.go.jp/). All ancestors of each cultivar were counted except for the recurrent parent, and we used this data to calculate the frequencies of the 11 most common ancestral landraces.

### Estimation of the degree of linkage disequilibrium

To compare the linkage disequilibrium (LD) among the domestic irrigated members of the three groups defined based on the three breeding phases, we estimated LD as the pairwise Δ^2^ between neighboring SNPs within a distance of 2000 kb. The numbers of irrigated accessions were 41 in Group 1, 50 in Group 2, and 63 in Group 3. The definition of Δ^2^ was equivalent to that of ρ^2^ [56] and was calculated using the formula described by Hill and Weir [57]. For each of the three groups, the mean complete LD (0<Δ^2^<1) was also calculated for each 50-kb range of intervals between adjacent SNPs (from 0 to 2000 kb).

### Haplotype analysis

Among the 12 rice chromosomes, we defined 3211 haplotype blocks based on consecutive alleles in five-SNP windows. The mean window size in the rice genome is estimated to be about 583 kb; that is, it equals (380 Mb/3259 SNPs)×5 SNPs. The diversity index for each of the 3211 haplotype blocks was calculated as [number of polymorphisms in haplotype blocks defined by SNP allele combinations in consecutive five-SNP windows (*N* = 3211)]/[number of landraces or cultivars in each group]. Diversity index values were compared between corresponding haplotype blocks located at the same position in each breeding group. We compared the diversity index values between groups using the Mann–Whitney test. We analyzed the association between the year of registration of an accession and the number of new haplotype polymorphisms by means of linear regression. All statistical analysis was performed using ver. 2.11.1 of the R software [58].

## Supporting Information

Figure S1 **Chromosomal distribution of SNPs used in our analysis of the genome structure of the Japanese rice population.** Vertical rectangles represent chromosomes 1 to 12 (from left to right) and colored horizontal bars indicate the locations of SNPs. Red arrowheads indicate the position of the centromere in each chromosome.
(PPT)

Figure S2 **Plot of the DIC value obtained using the InStruct software as a function of the** ***K*** **value.** Each point represents the average value for all accessions for a given *K* value. Five trials were carried out, with a burn-in cycle of 100 000 iterations followed by a further 200 000 iterations.
(PPT)

Table S1 **List of the cultivars used in the analysis.**
(XLS)

Table S2 **List of the 3259 SNPs used for the haplotype definition.**
(XLS)

Table S3 **The chromosomal positions of haplotype blocks for which groups 2 and 3 had less than half of the number of polymorphisms in Group 1.**
(XLS)

Table S4 **The 46 new polymorphic SNPs with alleles that were not detected in the Group 1 population.**
(XLS)

## References

[1] Hargrove TR, Cabanilla VL (1979). Impact of semi-dwarf varieties on Asian rice-breeding programs.. Bioscience.

[2] Peng S, Laza RC, Visperas RM, Sanico AL, Cassman KG (2000). Grain yield of rice cultivars and lines developed in the Philippines since 1966.. Crop Sci.

[3] Horie T, Shiraiwa T, Homma K, Katsura K, Maeda S (2005). Can yields of lowland rice resume the increases that they showed in the 1980s?. Plant Prod Sci.

[4] Yamamoto T, Nagasaki H, Yonemaru JI, Ebana K, Nakajima M (2010). Fine definition of the pedigree haplotypes of closely related rice cultivars by means of genome-wide discovery of single-nucleotide polymorphisms.. BMC Genomics.

[5] Yamasaki M, Wright SI, McMullen MD (2007). Genomic screening for artificial selection during domestication and improvement in maize.. Ann Bot.

[6] McNally KL, Childs KL, Bohnert R, Davidson RM, Zhao K (2009). Genomewide SNP variation reveals relationships among landraces and modern varieties of rice.. Proc Natl Acad Sci U S A.

[7] Zhao K, Wright M, Kimball J, Eizenga G, McClung A (2010). Genomic diversity and introgression in *O. sativa* reveal the impact of domestication and breeding on the rice genome.. PLoS One.

[8] Ebana K, Yonemaru JI, Fukuoka S, Iwata H, Kanamori H (2010). Genetic structure revealed by a whole-genome single-nucleotide polymorphism survey of diverse accessions of cultivated Asian rice (*Oryza sativa* L.).. Breed Sci.

[9] Wang L, Wang A, Huang X, Zhao Q, Dong G (2011). Mapping 49 quantitative trait loci at high resolution through sequencing-based genotyping of rice recombinant inbred lines.. Theor Appl Genet.

[10] Huang X, Wei X, Sang T, Zhao Q, Feng Q (2010). Genome-wide association studies of 14 agronomic traits in rice landraces.. Nat Genet.

[11] Huang X, Feng Q, Qian Q, Zhao Q, Wang L (2009). High-throughput genotyping by whole-genome resequencing.. Genome Res.

[12] Zhang Q, Maroof MAS, Lu TY, Shen BZ (1992). Genetic diversity and differentiation of *indica* and *japonica* rice detected by RFLP analysis.. Theor Appl Genet.

[13] Kono I, Takeuchi Y, Shimano T, Sasaki T, Yano M (2000). Comparison of efficiency of detecting polymorphism among *japonica* varieties in rice using RFLP, RAPD, AFLP and SSR markers.. Breeding Res.

[14] Nagasaki H, Ebana K, Shibaya T, Yonemaru Ji, Yano M (2010). Core single-nucleotide polymorphisms - a tool for genetic analysis of the Japanese rice population.. Breed Sci.

[15] Arai-Kichise Y, Shiwa Y, Nagasaki H, Ebana K, Yoshikawa H (2011). Discovery of genome-wide DNA polymorphisms in a landrace cultivar of *japonica* rice by whole-genome sequencing.. Plant Cell Physiol.

[16] Gao H, Williamson S, Bustamante CD (2007). A Markov chain Monte Carlo approach for joint inference of population structure and inbreeding rates from multilocus genotype data.. Genetics.

[17] Yonemaru Ji, Yamamoto T, Fukuoka S, Uga Y, Hori K (2010). Q-TARO: QTL annotation rice online database.. Rice.

[18] Yamamoto T, Yonemaru J, Yano M (2009). Towards the understanding of complex traits in rice: Substantially or Superficially?. DNA Res.

[19] Feng L, Wang K, Li Y, Tan Y, Kong J (2007). Overexpression of SBPase enhances photosynthesis against high temperature stress in transgenic rice plants.. Plant Cell Rep.

[20] Fukuda A, Nakamura A, Tagiri A, Tanaka H, Miyao A (2004). Function, intracellular localization and the importance in salt tolerance of a vacuolar Na(+)/H(+) antiporter from rice.. Plant Cell Physiol.

[21] Furukawa T, Maekawa M, Oki T, Suda I, Iida S (2007). The *Rc* and *Rd* genes are involved in proanthocyanidin synthesis in rice pericarp.. Plant J.

[22] Itoh H, Tatsumi T, Sakamoto T, Otomo K, Toyomasu T (2004). A rice semi-dwarf gene, *Tan-ginbozu* (*D35*), encodes the gibberellin biosynthesis enzyme, *ent*-kaurene oxidase.. Plant Mol Biol.

[23] Kang H-G, Park S, Matsuoka M, An G (2005). White-core endosperm *floury endosperm-4* in rice is generated by knockout mutations in the C_4_-type pyruvate orthophosphate dikinase gene (*OsPPDKB*).. Plant J.

[24] Kong Z, Li M, Yang W, Xu W, Xue Y (2006). A novel nuclear-localized CCCH-type zinc finger protein, OsDOS, is involved in delaying leaf senescence in rice.. Plant Physiol.

[25] Kusaba M, Ito H, Morita R, Iida S, Sato Y (2007). Rice NON-YELLOW COLORING1 is involved in light-harvesting complex II and grana degradation during leaf senescence.. Plant Cell.

[26] Lee S-K, Hwang S-K, Han M, Eom J-S, Kang H-G (2007). Identification of the ADP-glucose pyrophosphorylase isoforms essential for starch synthesis in the leaf and seed endosperm of rice (*Oryza sativa* L.).. Plant Mol Biol.

[27] Matsui H, Miyao A, Takahashi A, Hirochika H (2010). Pdk1 kinase regulates basal disease resistance through the OsOxi1-OsPti1a phosphorylation cascade in rice.. Plant Cell Physiol.

[28] Monna L, Kitazawa N, Yoshino R, Suzuki J, Masuda H (2002). Positional cloning of rice semidwarfing gene, *sd-1*: rice “Green Revolution Gene” encodes a mutant enzyme involved in gibberellin synthesis.. DNA Res.

[29] Morita R, Sato Y, Masuda Y, Nishimura M, Kusaba M (2009). Defect in non-yellow coloring 3, an α/β hydrolase-fold family protein, causes a stay-green phenotype during leaf senescence in rice.. Plant J.

[30] Ogo Y, Kobayashi T, Nakanishi Itai R, Nakanishi H, Kakei Y (2008). A novel NAC transcription factor, IDEF2, that recognizes the iron deficiency-responsive element 2 regulates the genes involved in iron homeostasis in plants.. J Biol Chem.

[31] Ono E, Wong HL, Kawasaki T, Hasegawa M, Kodama O (2001). Essential role of the small GTPase Rac in disease resistance of rice.. Proc Natl Acad Sci U S A.

[32] Sasaki A, Ashikari M, Ueguchi-Tanaka M, Itoh H, Nishimura A (2002). Green revolution: a mutant gibberellin-synthesis gene in rice.. Nature.

[33] Sazuka T, Aichi I, Kawai T, Matsuo N, Kitano H (2005). The rice mutant *dwarf bamboo shoot 1*: a leaky mutant of the NACK-type kinesin-like gene can initiate organ primordia but not organ development.. Plant Cell Physiol.

[34] Song S-Y, Chen Y, Chen J, Dai X-Y, Zhang W-H (2011). Physiological mechanisms underlying OsNAC5-dependent tolerance of rice plants to abiotic stress.. Planta.

[35] Ueguchi-Tanaka M, Ashikari M, Nakajima M, Itoh H, Katoh E (2005). *GIBBERELLIN INSENSITIVE DWARF1* encodes a soluble receptor for gibberellin.. Nature.

[36] Xie K, Wu C, Xiong L (2006). Genomic organization, differential expression, and interaction of SQUAMOSA promoter-binding-like transcription factors and microRNA156 in rice.. Plant Physiol.

[37] Yano M, Katayose Y, Ashikari M, Yamanouchi U, Monna L (2000). *Hd1*, a major photoperiod sensitivity quantitative trait locus in rice, is closely related to the Arabidopsis flowering time gene *CONSTANS*.. Plant Cell.

[38] Zhou J, Jiao F, Wu Z, Li Y, Wang X (2008). *OsPHR2* is involved in phosphate-starvation signaling and excessive phosphate accumulation in shoots of plants.. Plant Physiol.

[39] Zhu Q-H, Hoque M, Dennis E, Upadhyaya N (2003). Ds tagging of *BRANCHED FLORETLESS 1* (*BFL1*) that mediates the transition from spikelet to floret meristem in rice (*Oryza sativa* L).. BMC Plant Biology.

[40] International Rice Genome Sequencing Project (2005). The map-based sequence of the rice genome.. Nature.

[41] Mather KA, Caicedo AL, Polato NR, Olsen KM, McCouch S (2007). The extent of linkage disequilibrium in rice (*Oryza sativa* L.).. Genetics.

[42] Agrama H, Eizenga G (2008). Molecular diversity and genome-wide linkage disequilibrium patterns in a worldwide collection of *Oryza sativa* and its wild relatives.. Euphytica.

[43] Jin L, Lu Y, Xiao P, Sun M, Corke H (2010). Genetic diversity and population structure of a diverse set of rice germplasm for association mapping.. Theor Appl Genet.

[44] Ashikari M, Sasaki A, Ueguchi-Tanaka M, Itoh H, Nishimura A (2002). Loss-of-function of a rice gibberellin biosynthetic gene, *GA20 oxidase* (*GA20ox-2*), led to the rice ‘Green Revolution’.. Breed Sci.

[45] Asano K, Takashi T, Miura K, Qian Q, Kitano H (2007). Genetic and molecular analysis of utility of *sd1* alleles in rice breeding.. Breed Sci.

[46] Asano K, Yamasaki M, Takuno S, Miura K, Katagiri S (2011). Artificial selection for a green revolution gene during japonica rice domestication.. Proc Natl Acad Sci U S A.

[47] Takahashi Y, Teshima KM, Yokoi S, Innan H, Shimamoto K (2009). Variations in Hd1 proteins, *Hd3a* promoters, and *Ehd1* expression levels contribute to diversity of flowering time in cultivated rice.. Proc Natl Acad Sci U S A.

[48] Lin HX, Yamamoto T, Sasaki T, Yano M (2000). Characterization and detection of epistatic interactions of 3 QTLs, *Hd1*, *Hd2*, and *Hd3*, controlling heading date in rice using nearly isogenic lines.. Theor Appl Genet.

[49] Lin H, Liang Z-W, Sasaki T, Yano M (2003). Fine mapping and characterization of quantitative trait loci *Hd4* and *Hd5* controlling heading date in rice.. Breed Sci.

[50] Uga Y, Nonoue Y, Liang ZW, Lin HX, Yamamoto S (2007). Accumulation of additive effects generates a strong photoperiod sensitivity in the extremely late-heading rice cultivar ‘Nona Bokra’.. Theor Appl Genet.

[51] Yamamoto T, Lin H, Sasaki T, Yano M (2000). Identification of heading date quantitative trait locus *Hd6* and characterization of its epistatic interactions with *Hd2* in rice using advanced backcross progeny.. Genetics.

[52] Ebana K, Shibaya T, Wu J, Matsubara K, Kanamori H (2011). Uncovering of major genetic factors generating naturally occurring variation in heading date among Asian rice cultivars.. Theor Appl Genet.

[53] Fujino K, Wu J, Sekiguchi H, Ito T, Izawa T (2010). Multiple introgression events surrounding the *Hd1* flowering-time gene in cultivated rice, *Oryza sativa* L.. Mol Genet Genomics.

[54] Yamamoto R, Kushibuchi K (1992). Transition and prospects of rice breeding.;.

[55] Perrier X, Jacquemoud-Collet JP (2006). http://darwin.cirad.fr/darwin.

[56] Devlin B, Risch N (1995). A comparison of linkage disequilibrium measures for fine-scale mapping.. Genomics.

[57] Hill WG, Weir BS (1994). Maximum-likelihood estimation of gene location by linkage disequilibrium.. Am J Hum Genet.

[58] R Development Core Team (2005). R: A language and environment for statistical computing.. http://www.R-project.org.

