# Supplementary material for: Genome-Wide Haplotype Changes Produced by Artificial Selection during Modern Rice Breeding in Japan
Source: PLoS One. 2012 Mar 13;7(3):e32982. doi: 10.1371/journal.pone.0032982 (PMC3302797; doi:10.1371/journal.pone.0032982)
Supplement: Figure S1 — Chromosomal distribution of SNPs used in our analysis of the genome structure of the Japanese rice population. Vertical rectangles represent chromosomes 1 to 12 (from left to right) and colored horizontal bars indicate the locations of SNPs. Red arrowheads indicate the position of the centromere in each chromosome. (PPT) [file pone.0032982.s001.ppt]

## Slide 1
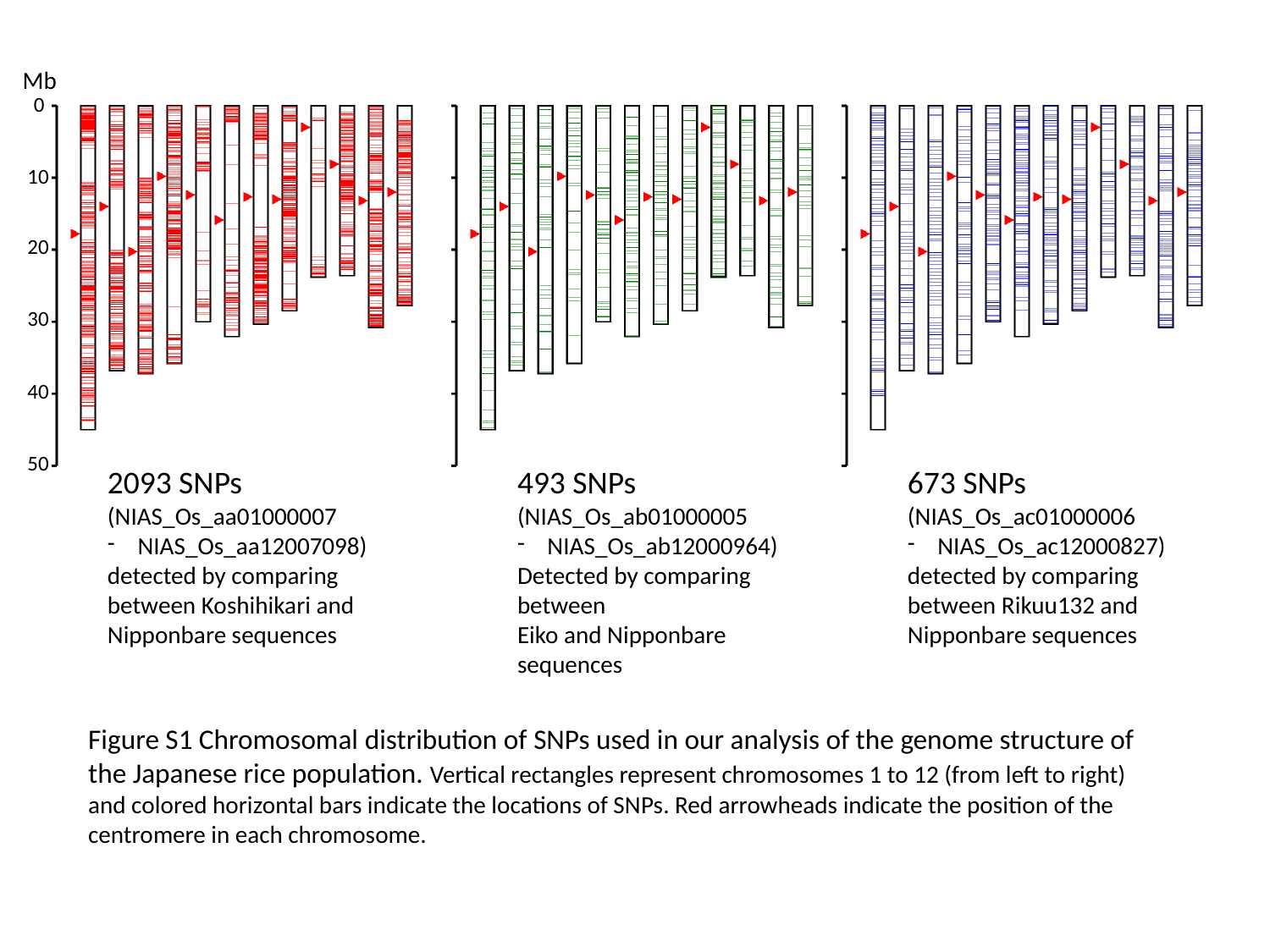

Mb
0
10
20
30
40
50
2093 SNPs
(NIAS_Os_aa01000007
NIAS_Os_aa12007098)
detected by comparing between Koshihikari and Nipponbare sequences
493 SNPs
(NIAS_Os_ab01000005
NIAS_Os_ab12000964)
Detected by comparing between
Eiko and Nipponbare sequences
673 SNPs
(NIAS_Os_ac01000006
NIAS_Os_ac12000827)
detected by comparing between Rikuu132 and Nipponbare sequences
Figure S1 Chromosomal distribution of SNPs used in our analysis of the genome structure of the Japanese rice population. Vertical rectangles represent chromosomes 1 to 12 (from left to right) and colored horizontal bars indicate the locations of SNPs. Red arrowheads indicate the position of the centromere in each chromosome.
