# Supplementary material for: Genome-Wide Haplotype Changes Produced by Artificial Selection during Modern Rice Breeding in Japan
Source: PLoS One. 2012 Mar 13;7(3):e32982. doi: 10.1371/journal.pone.0032982 (PMC3302797; doi:10.1371/journal.pone.0032982)
Supplement: Figure S2 — Plot of the DIC value obtained using the InStruct software as a function of the K value. Each point represents the average value for all accessions for a given K value. Five trials were carried out, with a burn-in cycle of 100 000 iterations followed by a further 200 000 iterations. (PPT) [file pone.0032982.s002.ppt]

## Slide 1
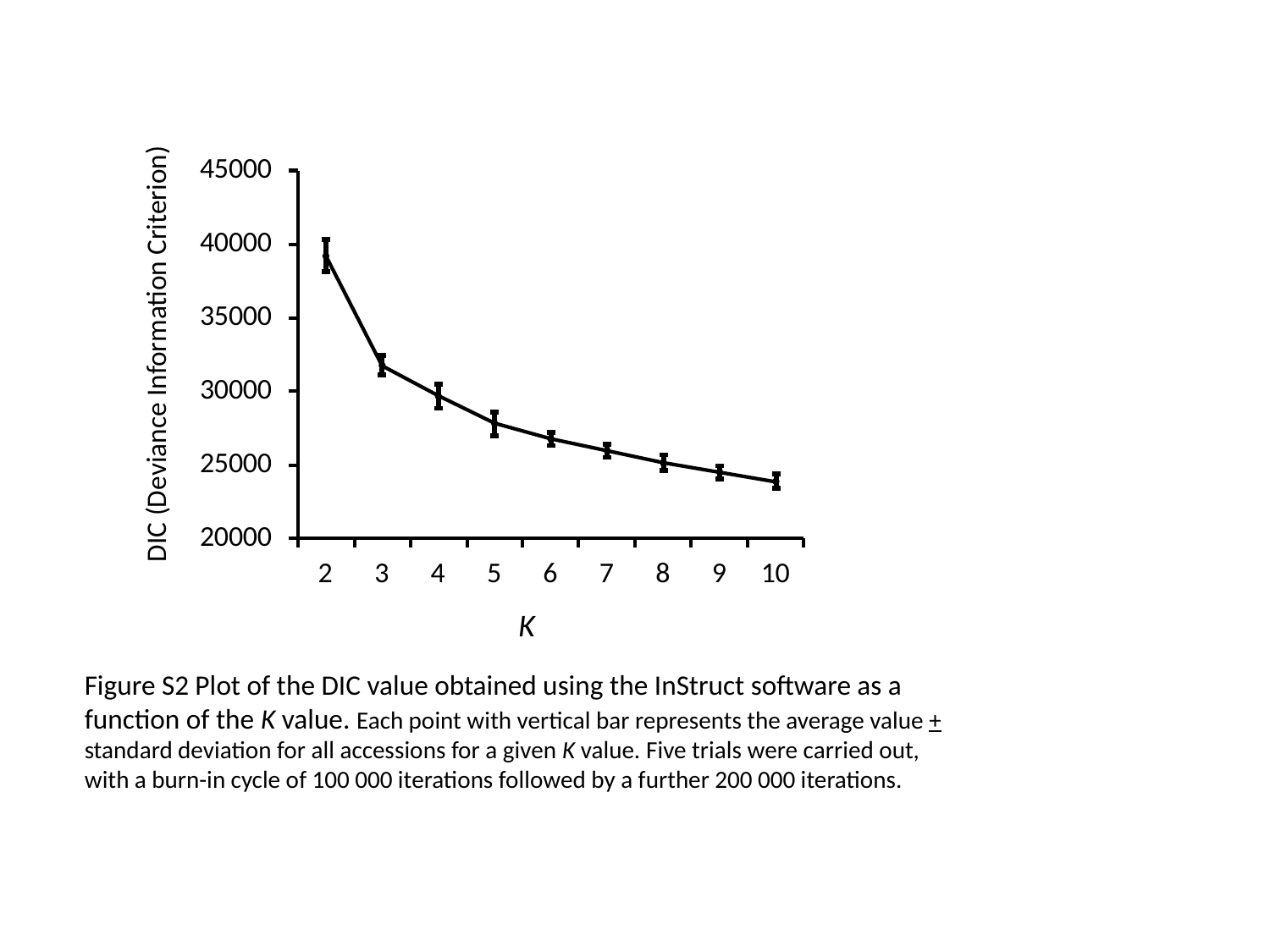

DIC (Deviance Information Criterion)
K
Figure S2 Plot of the DIC value obtained using the InStruct software as a function of the K value. Each point with vertical bar represents the average value + standard deviation for all accessions for a given K value. Five trials were carried out, with a burn-in cycle of 100 000 iterations followed by a further 200 000 iterations.
